# Injury and illness surveillance monitoring in team sports: a framework for all

**DOI:** 10.1186/s40621-024-00504-6

**Published:** 2024-06-10

**Authors:** Bradley Sprouse, Avinash Chandran, Neel Rao, Adrian J. Boltz, Molly Johnson, Philip Hennis, Ian Varley

**Affiliations:** 1 Nottingham Trent University, Nottingham, UK; 2 Datalys Center for Sports Injury Research and Prevention, Indianapolis, IN USA; 3 Michigan Concussion Center, University of Michigan, Ann Arbor, MI USA

**Keywords:** Injury, Illness, Epidemiology, Monitoring systems, Framework, Barriers, Data collection

## Abstract

**Background:**

Sport-related injuries and illnesses can negatively impact athlete welfare at all standards of participation in team sports. Injury and illness surveillance (IIS), and the development of monitoring systems, initiates the sequence of injury and illness prevention. Operational IIS monitoring systems help to appraise epidemiological estimates of injury and illness incidence and burden in various athlete populations. However, the methodological underpinnings of various monitoring systems are not harmonized or widely documented, with the presence of efficient and successful programmes rarely showcased at non-elite levels. The aim is to provide a framework that guides the development of IIS, which will enhance overall surveillance, to indirectly inform injury prevention strategies.

**Methods:**

The process involved all members of the research group initially discussing the research gaps, scope of the project, and the aims of the article. Unique experiences were shared, and specific and global challenges and barriers to IIS at all standards of team sport participation were identified. A tiered system of data collection with corresponding content were produced, with experiences and guidance provided throughout the article.

**Results:**

The literature has been reviewed and using first-hand experience in conducting IIS programmes in complex and diverse sport settings, the authors have identified key enablers and barriers for best practise as time, technological and human resources, reporter/practitioner training, and medical expertise. Areas of greatest importance regarding the conducting of IIS have been outlined, providing guidance and recommendations across all levels of team sport participation. These areas include definitions, data context, collection procedures, handling, security, ethics, storage, dissemination, quality, compliance, and analysis. Given the barriers to IIS, 3-tiered levels of data collection and content have been proposed. The levels indicate data collection variables, with a focus on sufficiency and achievability, aiming to support the successful conducting of IIS in team sports across all standards of participation. Future opportunities in IIS have been discussed, with several predictive measures and analytical techniques expanded upon.

**Conclusions:**

The framework provides universal guidance for implementing IIS monitoring systems, facilitating athletes, coaches, parents/guardians, governing bodies and practitioners to implement IIS processes, identify challenges, complete analysis, and interpret outcomes at all standards of participation.

**Supplementary Information:**

The online version contains supplementary material available at 10.1186/s40621-024-00504-6.

## Background

Sport-related injuries and illnesses can negatively impact individuals’ quality of life (Tremblay 2018). Injury and illness surveillance (IIS), and the development of injury and illness monitoring systems, is the vital first step in maintaining and enhancing player welfare in sport (Finch 2006). IIS initiates the sequence of injury and illness prevention, firstly, by identifying associated key internal and external risk factors (Mechelen et al. 1992). The data captured by IIS monitoring systems implemented within large samples can be used to establish the incidence and burden of injury and illness to develop and subsequently inform etiological hypotheses for injury and illness risk (Chandran et al. 2019), gauging changes in factors such as exposure time, standard of play and rule changes. The data captured within monitoring efforts can then be utilised to target specific paradigms for further research, prevention strategies and interventions (Finch 2006; Chandran et al. 2019).

The effectiveness of injury and illness prevention strategies can only be validated if an operational IIS programme is in place pre- and post-intervention. The establishment of injury incidence estimates, and the subsequent interpretation of the results, enables team sport medical practitioners and support staff to understand the influence of competition and training. Moreover, players and parents/guardians must be made aware of the risk of participation. This emphasises the need for publicly available data that is disseminated and interpreted accurately from reliable sources, highlighting the vital role of sporting organisations, medical practitioners, and coaching staff, where applicable. Only then can the relative risk of participation be assessed. Parents/guardians of children and young athletes can use data from sports IIS monitoring systems to guide their children’s sporting choices, particularly if the child has a pre-existing underlying condition. Taken together, this informed decision making may indirectly influence the exacerbation of injuries or illnesses.

To conduct a reliable, accurate and operational IIS programme and develop IIS monitoring systems to determine these estimates, standardisation of methods, data collection schemes and analytical approaches are required, enabling direct comparisons to be made with known statistics. Several research groups have published consensus statements for the collection and analysis of sports injury and illness data in elite team sports such as football (Fuller et al. 2006; Waldén et al. 2020), rugby union (Fuller et al. 2007), rugby league (King et al. 2009), and cricket (Orchard et al. 2016). In 2020, the International Olympic Committee (IOC) formed an updated consensus statement providing recommendations on injury and illness data collection, generalised across sports, to encourage consistency and enable comparisons to be made within and between sports (Bahr et al. 2020). However, the ability to implement IIS in line with consensus statement guidelines may be a challenge for teams with limited technological and human resources and capacity to collect the data required. Despite this, there is little, high quality research evidence at community based, recreationally active/trained levels. Therefore, this leads to difficulties with interpreting IIS data collected in these settings, rendering the risk of participation unknown at non-elite standards. Moreover, there is a lack of universal guidance, and little identification and management of challenges and barriers faced when collecting IIS data. Consequently, these may inhibit even the initiation of IIS data collection as well as further interpretation of any data captured in these settings. Specifically, such guidance at non-elite competition including sub-elite, development phases, recreational, and amateur levels is sparse. Despite statements providing useful information for elite sporting populations (Bahr et al. 2020), the minimum requirement for an effective IIS programme is yet to be outlined, which may be useful for teams at different standards of participation and with different resource availabilities.

Individuals at non-elite levels might be aspiring to play professionally, working in other occupations, or completing their academic studies alongside their sporting endeavours. Therefore, a serious injury or illness might have a negative impact on their development into the elite game or hinder an individual’s every day working and social life. That said, given the immediate and long-lasting physical, psychological, and economic issues associated with injury in elite populations (Ekstrand 2013), this may have many negative implications for sporting and non-sporting aspects of life. The presence of efficient and successful IIS monitoring systems is rarely witnessed at non-elite levels. Several perceived challenges and barriers to IIS (e.g. time, resources) may impact successful execution at these levels. Although, with the introduction of IIS programmes, there is a possibility that the knowledge and expertise of researchers with experience of implementing IIS monitoring systems in elite standards of participation can be transferred into the non-elite. Therefore, customised information and guidance can be provided which may inform alterations in protocols, rules, guidance, funding, and support, benefiting teams regardless of standards.

Overall, there is a rationale for IIS monitoring systems to be established at all standards of team sport participation, with the aim of informing prevention efforts to indirectly aid in the enhancement of player welfare and athlete participation at all levels. To help achieve this, there is a need for universal and more detailed guidance to enable data collection, which can be gained from shared experiences from experts in the field of work. Therefore, the current article aims to provide medical practitioners, coaching staff, support staff, athletes, and parents/guardians across all levels of team sport participation with recommendations for implementing and adhering to IIS monitoring systems.

## Methods

Through a pre-established network, expert research groups with 50+ years of experience of IIS in high school, college, and professional team sports discussed the idea of an IIS framework for all. Four authors (BS, MJ, PH, IV) work predominantly in research conducted in professional team sports including, but not limited to, football, cricket, and lacrosse. Three authors (AC, NR, AB) work in research conducted in a variety of high school, college, and elite team sports. Three authors established the initial ideas for the project (IV, BS, AC), before expanding to the wider research group.

Firstly, the research group identified the research gaps, the research question and determined the goals and objectives of the current framework. Consequently, this informed the aim of the article, to provide a framework, guidance, and recommendations to medical practitioners, coaching staff, support staff, athletes, and parents/guardians across all levels of team sport participation for implementing and adhering to IIS monitoring systems.

Throughout the process, all members of the research group were given the opportunity to discuss and make suggestions on the scope and direction of the project. Upon agreement of the aims, roles and responsibilities of each research group member were established. Each member was sought to provide information to support data curation, storage, practitioner guidance, and outline the challenges and barriers associated with IIS monitoring systems. To achieve this communication channels and protocols for regular updates, feedback, and coordination were put in place, whereby discussions would be undertaken by at least three members of the research group. Collaboratively, a research plan and design were then developed, outlining the methodology, timeline, and milestones.

The research group comprehensively reviewed available consensus statements and frameworks (Fuller et al. 2006, 2007; Waldén et al. 2020; King et al. 2009; Orchard et al. 2016; Bahr et al. 2020; Brown et al. 2019), identifying data collection variables that have previously been recommended. Given the current article aims to provide a framework and recommendations across varying standards of team sport participation, the research group discussed the feasibility of collecting each variable. These discussions then facilitated the establishment of variables that were best suited for each level of team sport play to achieve relative success within IIS monitoring. Research group members were also individually interviewed to provide their unique experiences of IIS, highlighting specific and global challenges and barriers to IIS data collection and curation.

Based on these discussions, the aim was to create a framework for all surrounding:

- Most important areas of IIS monitoring
- Perceived challenges and barriers to IIS monitoring
- A tiered system of data collection with corresponding guidance

Following regular meetings, updates and obtaining feedback, a draft manuscript was produced and distributed to all research group members. Continual feedback, follow-ups and discussions then occurred. Throughout the drafting process, research findings were collaboratively interpreted, with careful considerations for their implications and significance continuously discussed. Results were synthesised to ensure the answering of the research question, and that aims were achieved. Regular notes and plans were collated throughout wider group meetings. The level of agreement was quantified by all research group members being confident with the proposal of new ideas, alterations, and inclusions/exclusions. Divergent perspectives were identified, and conflicting interpretations were reconciled through consensus-based discussions. The effectiveness of the methodology in achieving the research aims was consistently evaluated. It was the responsibility of the project leads (IV, BS, AC) to ensure all agreed before progressing through each stage of the process.

Updated draft manuscripts were continuously produced and distributed to gain further insight from research group members. Revisions were considered and accepted where applicable throughout the drafting process, until a final draft was agreed upon by all seven members. The general processes by which the current framework was produced have been briefly outlined in an additional file (Additional file 1).

## Results

As a result of discussions between the research group members, the ability to collect data for IIS purposes was separated into “Levels”. Based on the Participant Classification Framework, 6-tiers have been established (Tier 0: Sedentary; Tier 1: Recreationally Active; Tier 2: Trained/Developmental; Tier 3: Highly Trained/National Level; Tier 4: Elite/International Level; Tier 5: World Class; (McKay et al. 2021)). Based on these tiers, authors formulated a 3-tiered system of data collection for the purpose of IIS monitoring. The tiers are hereafter referred to as “Levels”, which correspond to the standard of participation and the perceived resources and capabilities which influence the ability to collect specific categories of IIS data. Briefly, Level 1 refers to Recreationally Active/Trained/Developmental settings, Level 2 refers to Sub-Elite settings, and Level 3 refers to Professional settings, with the third level encompassing all categories. A visual representation of the levels of data collection outlining the minimum requirements to begin to conduct an efficient IIS programme, as well as the progressive methodological processes that could enhance IIS practice is shown in Table 1.

**Table 1:** Proposed levels of data collection within injury and illness surveillance programmes

	Minimum information required to be able to undertake IIS					Additional information to enhance the IIS									Real-time surveillance			
Data Collection Level	Event	Date of Case	Date Returned	Diagnosis (Location/Type)	Exposure	Onset	Cause	Activity	Time	Recurrent	Surface	Side	Position	Diagnosis with OSIICS	Player Profiling	GPS/Wearables	Video Analysis	Match/Training Statistics
**Level 1**	Y	Y	Y	Y	Y													
**Level 2**	Y	Y	Y	Y	Y	Y	Y	Y	Y	Y	Y	Y	Y	Y				
**Level 3**	Y	Y	Y	Y	Y	Y	Y	Y	Y	Y	Y	Y	Y	Y	Y	Y	Y	Y

“Y” directly corresponds to the sets of variables that are to be considered for each data collection level, where Level 2 encompasses Level 1, and Level 3 encompasses both Levels 1 and 2

The following areas were identified as having greatest importance, requiring guidance and recommendations across all levels of team sport participation:

1. Definitions & context-specific data needs
2. Data collection procedures
3. Data handling, security, ethical considerations, data storage & dissemination
4. Data quality, compliance, analysis, interpretation & recommendations
5. Levels of data collection & content
6. Future opportunities in IIS

Based on experience and discussions across research groups, several challenges, and barriers that team sport medical practitioners, coaching staff, team support staff, athletes and parents/guardians may face when attempting to collect IIS data have been established. These include:

1. Time
2. Technological and human resources
3. Reporter/practitioner training
4. Medical expertise

Additionally, injury, illness, and exposure information are to be recorded and classified by team sport medical practitioners, coaching staff, team support staff, athletes, or parents/guardians. To standardise the methodological process and ensure efficient reporting, injury, illness, and exposure data collection sheets have been developed and customised for each Level, which have been included as additional files (Additional file 2, Additional file 3, Additional file 4).

The accurate collection and curation of team sport injury and illness data is crucial in identifying when, where, and how injuries and illnesses occur (i.e., match-play or training), as well as the classification of diagnoses and factors associated with the reported cases. Based on the combined experience of IIS researchers, the greatest challenges to universal IIS were time, technological and human resources, reporter/practitioner training, and medical expertise. As the current article has been produced for varying standards of team sport participation, it is understood that non-elite teams may not possess the infrastructure to employ medical practitioners. Therefore, the current article hopes to provide support to team sport coaching and support staff, athletes and parents/guardians with the collection, curation, and storage of IIS data.

To encourage good medical practice and ensure IIS is conducted to the highest possible standard at all levels of participation, the current article and additional files (Additional file 2, Additional file 3, Additional file 4) have been produced to outline the minimum requirements to begin to conduct an efficient IIS programme, as well as more progressive methodological processes that could enhance current practice in IIS. The authors hope that by providing the recommendations in the form of Levels, this will offer a solution to overcoming the barriers faced when conducting IIS, allowing for the recording of informative and useful data.

As the discussion progresses, it is important to emphasise that the first 3 sections relating to the areas identified by the research groups contains information and recommendations that are important for all to consider; this is regardless of a team’s standard of participation.

## Discussion

### Definitions & context-specific data needs

#### Injury, illness & exposure definitions

From the International Olympic Consensus Statement published in 2020 (Bahr et al. 2020), it is recommended that the standardised definitions are adhered to for all levels of team sport participation to allow for universal comparisons.

These are as follows:

- Injury is tissue damage or other derangement of normal physical function due to participation in sports, resulting from rapid or repetitive transfer of kinetic energy.

- Illness is a complaint or disorder experienced by an athlete, not related to injury.

Exposure can be defined as any club/team directed activity and should be separated between match-play/competition and team/individual training/practice including, for example, strength and conditioning or recovery sessions. Moreover, as discussed previously, it is important to acknowledge that not all injury and illness monitoring efforts will have the same capacity to capture or distinguish between these occurrences consistently. Therefore, it is crucial to establish guidelines that align with the available technological and human resources and staff expertise within a particular setting. For instance, as depicted in Table 1., in situations where clinicians are not routinely present, surveillance may involve documenting events as they transpire with only minimal details recorded (e.g., inciting event, date of onset, etc.). Conversely, with the presence of a medical provider, additional information can be documented (e.g., exposure, body part affected etc.). It is equally important to consider the ultimate use-case for the collected data when engaging in data collection efforts, whether this is simply documenting the occurrences and severity of injuries and illnesses within a given setting or incorporating the downstream analysis of injury risk.

### Data collection procedures

Given the varying standards of participation, and the perceived barriers to IIS associated with different standards, additional files (Additional file 2, Additional file 3, Additional file 4) have been produced by the current research groups to aid in the collection of injury and illness data if required. It must be stressed that guaranteeing the minimum requirements are undertaken is crucial in producing accurate and usable data.

To gauge the relative risk of injury in a team sport, it is important that cases sustained outside of formal match-play or training should not be included in any data collection. On the other hand, illnesses can be asymptomatic for a period, resulting in late presentations of symptoms. This makes it impossible to determine their transmission, leading to difficulties when ascribing illnesses to sport participation. Therefore, it is important to record illnesses that occur both during and outside of formal match-play or training. It is also recommended that data are actively recorded on a regular basis (i.e. daily basis, as the event occurs) to ensure accuracy of reporting. This may differ depending on the regularity of training and match-play. The retrospective collection and self-reporting of data may reduce accuracy and quality, negatively influencing the practical utility due to recall bias (Althubaiti 2016), and is therefore not advised. However, the research group have identified and acknowledge that there are challenges and barriers to IIS data collection procedures (i.e. time, technological and human resources, reporter/practitioner training, medical expertise), mainly associated with amateur-level sport. Therefore, where retrospective reporting is unavoidable, it is recommended that reporting is conducted within a limited number of days or weeks of when an injury or illness occurs rather than, for example, months.

Based on the consensus statement (Bahr et al. 2020), the definition of time-loss includes the recording of any injury or illness that is reported to be ≥ 1 day absent from participation and is the definition that is maintained by the current article. The current framework based on the consensus statements available have been established based on evidence showing that multiple definitions over time have led to differences in the data reported. More specifically, variations have naturally been shown to occur in incidence and burden figures when utilising time-loss (≥ 1 day absent) and medical attention definitions (all injuries including 0 days absent), or a combination of both. For example, within the same study in South African men’s professional soccer players, a match injury incidence of 24.8/1000h was reported, while the incidence of time-loss injuries was reported to be 16.5/1000h (Bayne et al. 2018). Another example showed a large difference in match injury incidence between two single season investigations utilising the same number of teams in the men’s professional soccer in the USA (Morgan and Oberlander 2001) and England (Jones et al. 2019), whereby a medical attention (Morgan and Oberlander 2001) and a time-loss (Jones et al. 2019) injury definitions were utilised. Such a difference may not have been anticipated; however, many factors can influence fluctuations such as geographical location and inevitable inter-team variability. Despite this, the investigations highlight differences that can occur when using different injury definitions and methodological considerations.

IIS reporters must be aware that different definitions can be utilised, and between-clinician variability can be present during data collection processes. In particular, this can occur where the reporter or clinician’s expertise vary (i.e. physiotherapist vs. coach/parent/guardian), leading to differences in injury classifications according to definitions and standards. Therefore, understanding various definitions and differences in data collection processes is required to ensure an alignment of methodologies when collecting and comparing current data to previously reported findings, allowing for valid inter- and intra-sport comparisons.

#### Collecting player exposure

While the reporting of injury and illness frequency and days lost is informative, this offers limited practical utility when comparing within team sports between standards of participation, as well as between team sports. The collection of all competitive match-play and all team-directed training exposure information is important to enable a standardised value to be calculated for which injury and illness cases are expressed in the form of incidence and burden. For injuries, this is commonly reported by the number of injuries and days absent per 1000 h of exposure. On the other hand, illnesses are complex and can have ambiguous onsets and inciting events, and as aforementioned, this makes it difficult to determine when they are transmitted. Hence, the reporting of “player days” (typically, number of days in the season), where the number of illnesses per 1000 player days is expressed, is recommended.

Across all team sports, it is recommended that exposure is recorded on an individual basis within a team, which can then be calculated on a squad basis (Bahr et al. 2020). However, as aforementioned, this is dependent on the use-case for the collected data and the resources available. If the objective involves downstream analysis of injury risk, greater attention may be given to collecting exposure data alongside injury data, as this is essential for obtaining reliable injury rates, risk, and burden estimates. These exposure data can also be collected on an event basis, aggregated, or approximated based on the available staff resources. For example, when a limited number of challenges and barriers are faced when collecting exposure data, more comprehensive collection procedures can be undertaken via detailed electronic record notes (i.e. Additional file 3, Additional file 4), as well as real-time surveillance. However, where more challenges and barriers are present, approximating at-risk-exposure time using squad/roster sizes and the approximate number of team events (e.g., practices, matches) during a season is encouraged by the framework. This would yield more comprehensive information compared to not collecting any exposure data at all and is recommended within previous literature (Brown et al. 2019). Alternatively, if the primary aim were solely to document the occurrences of injuries within a given setting, such information could be regarded as superfluous, and attention may be directed elsewhere; perhaps towards capturing supplementary contextual details pertaining to injury events*.*

### Data handling, security, ethical considerations, data storage & dissemination

To adhere to ethical considerations and recommendations, it is of paramount importance that individuals responsible for the collection and storage of data understand the confidential nature of IIS programmes. Whether the data curated is being used for research or not, it is the responsibility of individuals collecting the data to do so accurately and store securely to protect all associated with the programme. It is recommended that data should be collected anonymously, saved in password protected files on multiple devices, and backed-up regularly via cloud, server, and local storage. Furthermore, it is important for data to be stored and shared in line with the country of origin’s data protection criteria and data management laws, which should be sought out prior to the data collection.

Data storage options encompass a range of solutions catering to diverse needs. In the present context, options such as relational or NoSQL database solutions offer meaningful avenues for consideration. Relational databases offer structured storage and ability to handle complex queries and data relationships. Conversely, NoSQL databases offer a non-relational approach suitable for managing unstructured and semi-structured data at scale, delivering high scalability and performance. Comparable alternatives include columnar databases, which are equipped to handle high analytical workloads and can intake large data volumes. Ultimately, the data harmonization and storage solutions will also depend on many factors (i.e. available technological and human resources). Considering the scope and scale of operations pertaining to any IIS monitoring system, it is imperative to systematically make choices regarding data harmonization and storage, with the aim of safeguarding factors like security and fidelity. Data storage considerations have been comprehensively reviewed (Hassan et al. 2022), including protection, privacy, and challenges, whereby the considerations are maintained by the current framework.

Data sharing and transparency is also important to strengthen practice of exercise and sport-related research (Halperin et al. 2018) and may help to enhance IIS monitoring and subsequent research, influencing applied practice at all levels of team sport participation. Specific to IIS analysis, this involves ensuring that reporters/practitioners are aware of how to correctly and effectively showcase the data collected, whether this is through the use of reports, infographics, presentations or databases. For example, having access to and presented with normative injury incidence statistics and an associated sport-specific relative risk of participation at different levels can influence a decision to take part or continue in a sport. At present, coaches/athletes/parents/guardians/governing bodies/practitioners rely on researchers, mainly associated with elite level sport, to provide data which may provide little practical utility across many populations. Therefore, responsibly open sourcing data collected and encouraging data sharing across all levels of team sport participation can contribute to improvements in the scope of research from non-elite, youth and collegiate to the elite levels.

### Data quality, compliance, analysis, interpretation & recommendations

The successful conducting of IIS is dependent upon the infrastructure and resources available to the team sports clubs, athletes, and staff. As previously outlined, perceived barriers to IIS based on the authors’ accumulative experience over prolonged periods have been identified. These barriers are potential hinderances for the collection of IIS data for some teams, particularly at a non-elite level, resulting in reduced data quality and compliance. In the pursuit of consistent data accuracy, and completeness, team sport medical practitioners, coaching staff, and team support staff must be trained and advised on data collection protocols and methodologies (Dreyer et al. 2019). Therefore, the authors recommend the introduction of short courses in IIS at all standards of participation which is implemented by governing bodies. Essentially, a member of staff affiliated with a team is obligated to undertake the relevant training, select the most achievable level of data collection, and collect the data. Similarly, the ability to attain certain data analysis practices and standards may differ depending on restrictions to data collection processes in various settings. For instance, without capturing data on at-risk exposure time or time spent in population, epidemiological estimates of injury metrics cannot be calculated. Given the challenges to data collection that are associated with variations in medical expertise and technological and human resources, it is important to align the data collected with plausible analysis. Hence why, for example, exposure is recommended to be collected within the framework (Level 1 of data collection, Table 1.). This can improve knowledge of injury and illness commonality and burden, gaining context and increasing the practical utility of the information. This allows for further analysis and comparisons to be made to previously published data on a team- and sport-specific basis, across varying standards of participation.

Within the range of any data collection operation, it is important to ensure quality and completeness of reporting, without a high proportion of missing data. Therefore, updating and quality control from a data collection and processing perspective plays a vital role in reviewing the data, and improving the accuracy and validity of further analysis. Once data collection processes have been undertaken, the implementation of active “follow-ups” for auditing against systematic standards is recommended. Specifically, inter-, and intra-reporting quality checks can ensure data has been collected as efficiently as possible despite the constraints teams may have at different standards of participation. One of which includes the determination of specific injury and illness types and diagnoses, which is considered to pose the most difficulty when reporting cases across varying populations (Brown et al. 2019). Therefore, it is important for those given the responsibility of collecting data to do so in as much detail as possible, which then enables later updates should the diagnoses be made clearer with time. It is important to emphasise that the higher the level of data collection that teams can attain, the more universally comparable the data will be, allowing for more in-depth and impactful conclusions and subsequent actions. Although, teams associated with the first level of data collection must be assured that their efforts to continuously collect this information will still provide information that can contribute to the aim of reducing the incidence and burden of injuries and illnesses within their team.

Although detailed consensus statements are widely accepted, anecdotal information suggests some aspects of an IIS programme continue to pose dilemmas when recording cases. For example, determining when a player has resumed to “normal” training or match-play to evaluate recovery of a time-loss case. Moreover, reporting the return date from a gradual onset and/or recurring case has also presented some difficulties given the ongoing nature of the case; both may lead to over-/under-estimations of severity. Methods for modelling and analysing time-loss that can incorporate injury severity and other individual injury related factors have previously been reported (Chandran et al. 2020). However, given the applied nature of IIS programmes, some of the challenges faced are inevitable and difficult to control. Therefore, certain aspects must be seen as a limitation, where an element of acceptance and flexibility is required to maximize the utility of the data collected regardless of the standard of participation.

The categorisation of injuries and illnesses during the reporting process is associated with challenges. For example, multiple injuries can occur via the same inciting event. Therefore, if an IIS monitoring system and ethical considerations allows for the determination of multiple injuries per event, and identifies the individual obtaining certain injuries, then reporting in this capacity can take place. However, should these processes present barriers for data collection in this manner, recording of the most serious case is recommended. For example, should a player suffer a compound fracture of the lower leg, it is likely that ligament damage will also occur. However, in this case, only the fracture would be recorded. Guidance has also suggested that data is to be grouped by anatomical type (i.e., contusion, muscle, ligament, fracture) and location (i.e., knee, ankle, lower leg, thigh) (Bahr et al. 2020). However, while current categorisation is informative, this arguably reduces the practical utility of the information. For instance, the incidence and severity of a quadricep haematoma will be different to that of muscle tear. Therefore, the introduction of more specified case diagnoses aims to provide more in-depth information to further support reduction of injury and illness incidence and severity; provided the resources exist to capture the information at this level of granularity.

The influence that successful IIS programmes can have within team sport is significant and widely recognised (Finch 2006). Additionally, the concept of data quality is of great importance. The data collected in single team, single season investigations may appear significantly variable when comparisons are made, reducing ecological validity. For example, in men’s professional domestic soccer in China, a match injury incidence of 61.1 injuries per 1000 h was reported (Lee et al. 2014), meanwhile in Holland a match injury incidence of 32.8 injuries per 1000 h was reported (Stubbe et al. 2015), which is considerably lower despite both being conducted over a single season, in less than 10 teams, using similar methods. The geographical location and style of play, which can be country dependent, may have contributed to this difference. Although, even within single populations, seasonal variations can often be reported. For example, seasonal variations in match injury incidence were observed in English International teams across 8 years of data collection (Sprouse et al. 2020). Therefore, such variations provide an added argument for data sharing, and multiple seasons of data collection are required to facilitate aggregated analyses and elucidation of varying sources of heterogeneity that can be associated with single team, single season investigations.

### Levels of data collection & content

#### Level 1—recreationally active/trained/developmental

Within Level 1, it is likely that coaches, athletes, parents/guardians will be tasked with reporting IIS information on behalf of the players. It has previously been reported that medical staff (e.g. ATs, physiotherapists) are better positioned to report IIS data than coaches and parents/guardians (Yard et al. 2009). It is acknowledged that the teams associated with this level may not possess the capability of employing medical staff. Therefore, all the suggested challenges and barriers outlined in the results apply to teams corresponding with Level 1. To combat these, a layered approach to data collection has been suggested in the current framework, where Level 1 has been established by identifying the minimum requirements for a successful IIS programme to be conducted. It has been reported that shorter surveys and questionnaires result in a higher response rate from participants (Edwards et al. 2002). Therefore, it is feasible to suggest that complex, time consuming data collection processes for IIS may lead to a lack of compliance. Current authors experience suggests that a barrier to implementing IIS monitoring systems is technological resources. Thus, the collection of injury and illness data can be more challenging without an electronic medical record system (EMR) in place, with the process of collecting exposure data most cumbersome given the inability to utilise GPS monitoring devices and wearables.

Level 1 and the corresponding additional files (A2 File, A3 File, A4 File) containing data collection sheets have been produced for coaches, athletes, parents/guardians, and teams with little to no medical support staff to provide the most minimalistic but optimal methods for successful data collection, whilst helping to maximise the perceived limited time and technological and human resources available at community-based standards of participation.

Identifying which events injuries and illnesses occur in, whether this is during match-play or training, enables subsequent analysis to distinguish between the two events, given that it has been widely reported that match-play has a higher injury risk than training in a variety of professional team sports (Sprouse et al. 2020; Mack et al. 2020), and youth/collegiate team sports (Chandran et al. 2021a, 2021b; Lempke et al. 2021; Wasserman et al. 2019; Pierpoint et al. 2019). Additionally, the reporting of accumulated time lost enables severity of injuries and illnesses to be identified. The reporting of these variables alongside estimates for total, match and training exposure allows for the calculation of incidence and burden per 1000 h or per 1000 player-days which provides a standardised measure; particularly burden which is now considered to be as additionally informative as incidence (Bahr et al. 2018). Moreover, the reporting of type and location of injuries and illnesses (where applicable) is also recommended within Level 1. As aforementioned, recreational level team sports will generally correspond with Level 1 of the 3-tiered system proposed in the current article. Based on this, the minimum level of detail for an injury suffered by a recreationally active basketball player, for example, would be the reporting of an ankle injury. This detail, simultaneously collected alongside other Level 1 variables, can help identify commonality and severity trends to determine those most burdensome to teams, thus requiring the greatest medical attention. Examples within research have included the identification of the ankle being the most common injury location across a combination of men’s and women’s, adult and youth, basketball (Andreoli et al. 2018), or the knee being most common in professional American football players (Mack et al. 2020). In the absence of this information, surface level statistics can only be reported (i.e. frequency of all injuries), rendering the understanding of specific injury trends and the implementation of prevention strategies more difficult.

The current framework has endeavoured to incorporate user-friendly injury type and location categories, and illness type categories which ensure the practical utility of information within an applied setting. Therefore, the categories outlined in previous consensus statements have formed the basis for those recommended in the current article, with the complete outline included as additional files (Additional file 2, Additional file 3, Additional file 4).

#### Level 2—sub-elite

As time and resources become more readily available to teams, it is likely that an individual(s) will be assigned to the collection and monitoring of information, which may include trained medical practitioners, increasing compliance and the ability to collect sufficient data required for the current level. Therefore, in addition to categories in Level 1, Level 2 also includes the collection of injury and athlete-specific characteristics including onset (i.e., acute or gradual), inciting event (i.e., contact or non-contact), activity (i.e., running, jumping/landing, tackling), side, recurrence, time, and surface. The current research groups agree that the categories of data collection incorporated in Level 2 could be perceived to be more difficult to report for teams with limited time and resources, particularly where information is reported retrospectively and is reliant on player recall. Hence, they have not been incorporated into Level 1.

The categories of data collection in Level 2 provide an element of explanation and useful context for the frequency, severity and type of injuries and illnesses upon further analysis. Within this, when establishing IIS monitoring systems, the worthy inclusion of diagnostic coding systems (i.e. multi-sport Orchard coding system (Orchard et al. 2010)) have been introduced to attach identifiers to injuries and illnesses to combat non-differential misclassification which arises via between-reporter variability, enhancing data harmonization and consistency between sports. Diagnostic coding systems strive to standardise reporting practices, yet between-reporter variability is not uncommon, with reporter/practitioner training potentially still presenting as a barrier to IIS monitoring. However, the information provided through standardised practice in Level 2 can continue to identify quantifiable trends. For example, observing more injuries occurring acutely in men’s English international soccer due to the physical nature of the sport (Sprouse et al. 2020), and identifying the most inciting activity (excluding general play and unknowns) for injury in men’s American football as blocking (Chandran et al. 2021c), or rebounding in women’s basketball (Lempke et al. 2021). This is applicable to previous or recurrent injuries, which have been reported to be one of the most common injury risk factors (Emery et al. 2005; Hägglund et al. 2006), or identifying susceptibility to injury based on surface (Gould et al. 2023). Furthermore, associations can be made between playing position and injury. For example, in American football, while more match injuries are reported in defensive secondaries and offensive linemen, match injury rates are higher in running backs, wide receivers, and tight ends (Mack et al. 2020).

An additional category to Level 2 also includes a more detailed approach through the reporting of diagnoses. An example of the minimum level of detail for an injury report by teams who correspond with Level 2 may be a sub-elite American football player, whose position is quarterback, and has suffered a knee medial collateral ligament injury via acute and contact mechanisms after a tackle. By reporting injuries or illnesses in this manner, it offers progression to subsequent analysis surrounding the information provided for type and location, identifying specific diagnoses that are having a negative affect and require greatest attention. Some examples within research include hamstring injuries which are reported to be common in field-based team sports (Maniar et al. 2023), with for instance a high rate documented in soccer which has increased over time (Ekstrand et al. 2023). In relation to injury burden, ACL injury has been reported to have a high burden in team sports such as soccer and American football (Mack et al. 2020; Horan et al. 2022). Concussions are also reported to be relatively common across both professional and many collegiate team sports settings (Gardner et al. 2019; McGroarty et al. 2020; Mooney et al. 2020; Mack et al. 2021; Chandran et al. 2022). Alternatively, the reporting of specific injuries may also shed a positive light on encouraging trends that have been identified. For example, recently it has been shown that the incidence of game concussions per season in American Football (National Football League, NFL) have decreased from 2015 to 2019, attributed to concussion reduction strategies introduced before 2018 (Mack et al. 2021). Information such as this is invaluable to players, coaching staff, medical practitioners and governing bodies to provide evidence that good medical practice, and any alterations implemented (i.e. medical support, rule changes, equipment upgrades), are potentially contributing to the reduction of specific injuries.

Ultimately, via the information provided across both Levels 1 and 2, this allows for the determination of more insightful and practically usable information, where medical practitioners have the time and experience to do so. It is important to note that should technological and human resources, and time, not be considered as barriers for teams generally associated with Level 1 (*Recreationally Active/Trained/Developmental*), it is advised that the data for the categories outlined in Level 2 are attempted to be collected where possible.

#### Level 3—professional

In addition to the Levels 1 and 2, Level 3 relies on the ideal situation where an experienced medical practitioner(s) attached to larger, more elaborate medical staffing departments, is assigned to successfully collect the data required to inform applied practice. Additionally, it is emphasised that data quality and completeness is of paramount importance to act on the data collected. It is understood that the categories outlined in the current level can be difficult to attain data for given the barriers to IIS previously outlined, particularly at lower standards of participation, so have therefore been included in Level 3. Ultimately, the additional technological and human resources potentially available at this level could allow for more granular injury, illness and exposure data collection and examinations that may not be feasible at other levels.

Due to rapid technological advancements and well-staffed departments, the ability to monitor players on an individual basis has become popular and more achievable in recent times. This has led to an ability to hypothesise cause of injury and illness in addition to regular surveillance. The use of wearable technology such as GPS, and the derived variables collected (i.e., high speed running (HSR), sprint distances, accelerations/decelerations, ACWR—acute:chronic workload ratio) has enabled associations to be made with between workload and injury risk. For example, an association between the ACWR and non-contact injury in team sports has been widely cited (Griffin et al. 2020). Also, a link between individual workload variables and heightened injury risk have been reported where high numbers of accelerations over 3 weeks have been associated with overall and non-contact injury risk in elite youth football (Bowen et al. 2017), as well as the tissue damage and fatigue caused by decelerations increasing sport-related injury risk where there is an inability to dissipate braking loads (McBurnie et al. 2022). These are just some examples of how IIS information can then lead to the hypothesised causes of injury and recommendations. The conclusions can then be applied to the assessment and training for specific workload variables, ultimately having a positive impact on player’s physical condition and injury prevention (Mechelen et al. 1992; Chandran et al. 2019).

Additionally, the creation of player profiles via medical screening, video analysis and recording of match/training statistics to determine overall loading on players has contributed to the identification of those at heightened risk of injury and/or illness. This has created scope to expand on the data collection requirements of current consensus statements, with the aim of enhancing IIS to inform more complex analytical processes through the assessment of individuals’ overall profiling status. An example within applied team sport is the recurrence of running related hamstring injuries within professional football. The use of video analysis and GPS, alongside other data collection variables outlined in Levels 1 and 2, may enhance the ability to understand the stimulus of a given injury. Associations with aspects of running (i.e. accelerations/decelerations), as well as weaknesses potentially identified during profiling and screening tests, may help to indirectly inform prevention strategies and applied practice.

It is also important to reiterate the need for data sharing at this level. Although useful to a certain extent, there is limited universal practical utility to information provided from a single team. Intra-league and sport comparisons are required to have certainty in the methods behind the data being collected, alongside notifying the trends, differences and avoiding erroneous spikes in data which may result in inefficient or misplaced policy solutions.

### Future opportunities for IIS

Prior to incorporating future supplementary data modules and inputs, it is important to consider regular audits and updates of existing processes and data elements as a fundamental aspect of maintaining IIS monitoring systems. The revision of existing processes and established standards not only facilitates timely enhancements to the system but also provides opportunities to align data elements with evolving clinical guidelines or consensus statements. Integrating this process into a routine cadence and encompassing it as part of codebook updates may ensure the continuous refinement and adaptation of IIS monitoring systems.

Given the rapid technological advancements that have been made within elite team sport, there is scope for more in-depth analyses to be conducted, expanding on the data collection requirements of current consensus statements. It is feasible to suggest that IIS analyses may begin to identify predictive measures for the onset of injuries and illnesses through the ability to collect “real-time surveillance” on an individual and team basis via the incorporation of technology. Ultimately, the information could be integrated via advanced analytical techniques such as modelling (i.e., machine learning (Eetvelde et al. 2021)) and digital twin simulations which aim to contextualize characteristics and be indicative predictors of future injury and illness (i.e. occurrence, severity, recovery). Such advanced analytical techniques have previously been utilised (Chandran et al. 2022), where concussion symptom presentation patterns were used to predict symptom resolution time in amateur athletes (National Collegiate Athletic Association, NCAA). Alongside contextual predictors, greater counts of specific symptoms were associated with longer symptom resolution time in this work (Chandran et al. 2022).

Meanwhile, machine learning has been successfully utilised for the prediction of sport-related injuries (Eetvelde et al. 2021). An accuracy of 85% was reported based on anthropometric, motor coordination and physical performance outcomes when predicting injury in elite youth soccer players (Rommers et al. 2020), and a high sensitivity and specificity of 77.8% and 83.8% were reported based on pre-season screening tests in professional soccer players (Ayala et al. 2019). Therefore, it is possible that future predictive methods using machine learning can be further improved by large volumes of data and varying types of data being collected (Verhagen and Bolling 2015). Moreover, it is feasible to suggest that the collection of GPS variables via sensors and wearables, video analysis and match/training statistics may provide the foundations for processes which may identify injury and illness predictors. Using data collected via a combination of the categories outlined in levels 3 and 4 (Table 1.), perceived optimal conditions for individuals can be tested via a virtual representation before they are implemented.

Physiological biomarkers, including protein fluid biomarkers, radio-imaging, and cardiovascular physiology indices could also be integrated into IIS in the future. Many candidate biomarkers have been explored (Lee et al. 2017), attempting to identify associations with athlete health, performance, and recovery. More specifically, regarding injuries and recovery, relevant biomarkers are likely to be injury specific. For example, target biomarkers investigated in brain injury/concussion (e.g. cell biomarker S-100 calcium binding protein B (S-100B), (Graham et al. 2011; Shahim et al. 2014)) are different in comparison to a ligament injury, such as ACLs (i.e. collagen type I and type II cleavage products, (Svoboda et al. 2013)). However, although there is promise being shown in a number of biomarkers being investigated (Lee et al. 2017), there is no consensus on the optimal biomarkers to target and monitor, with little clinical diagnosis. Nonetheless, reliable, and valid biomarkers have the potential to be used as tools for injury and illness mitigation in future IIS programmes.

That said, there are potential barriers associated with the implementation of more advanced analytical approaches. Similar to that of “surface-level” IIS, ethical considerations, data privacy, storage and dissemination may pose a challenge when attempting to utilise these methods. When attempting to introduce more predictive measures, or making associations between biomarkers and athlete health, more personal data is used to inform conclusions. The more data collected and analysed on behalf of athletes, and the more invasive the techniques become, this increases the personal and individualised nature of the approaches. Therefore, more emphasis is then placed on ethical considerations, given the invasive techniques potentially being utilised. Also, adherence to data privacy and storage policies to maintain confidentiality, anonymity, and data specifications also become of paramount importance (Hassan et al. 2022), as previously outlined in the “Data Handling, Security, Ethical Considerations, Data Storage & Dissemination” section of the current article. Additionally, such approaches will be associated with an increased cost, alongside a greater demand for resources and expertise to conduct complex methodologies that come with this. Therefore, although future opportunities further enhance IIS, more advanced analytical approaches favour the elite team sport populations where accessibility is more feasible.

Regardless of the current barriers that may hinder the implementation of future opportunities in IIS, a proactive approach to IIS monitoring via sophisticated analytical approaches can lead to the identification of thresholds, and may have a greater influence on the indirect enhancement of injury and illness prevention in the future.

## Conclusions

The current framework outlines challenges associated with IIS and provides recommendations, based on the collective research experience, to team sport medical practitioners, coaching staff, team support staff, as well as parents/guardians and athletes across all standards of team sport participation to aid in the collection of injury and illness surveillance data. This includes sub-elite competitions, development phases, collegiate, recreational, and amateur standards, as well as providing support at elite/professional levels. At sub-elite standards of team sport participation, where technological and human resources and medical expertise are limited, it may not be feasible to collect data to the standard of consensus statements. The current article and additional files (Additional file 2, Additional file 3, Additional file 4) allow for the introduction of impactful IIS via easy and time-efficient data collection processes. The negative consequences of injuries and illnesses that are suffered at sub-elite standards may vary to the elite/professional settings, impacting individuals at a sub-elite standard by preventing professional development, or affecting everyday working lives (i.e., occupational, academic). The conducting of an IIS programme has substantial benefits for all, providing strong foundations for subsequent analyses to take place, identifying trends, associations to risk factors and making comparisons to previously known statistics. Aiding the enhancement of overall surveillance can indirectly inform prevention interventions, for example, altering training and recovery programmes, monitoring individual and team loads and wellness, and influencing lifestyle changes. Ultimately, this may contribute to the maintenance and improvement of athlete welfare.

### Supplementary Information

**Additional file 1**. Workflow diagram

**Additional file 2**. Data collection sheets (Level 1 and 2)

**Additional file 3**. Monthly exposure data collection sheets (Recommended Level 1 and 2)

**Additional file 4**. Weekly exposure data collection sheets (Recommended Level 2 and 3)

## Data Availability

Data sharing is not applicable to this article as no datasets were generated or analysed during the current study.

## References

[CR1] Althubaiti A. Information bias in health research: definition, pitfalls, and adjustment methods. J Multidiscip Res. 2016;211–7. 10.2147/JMDH.S104807 PMC4862344 27217764

[CR2] Andreoli CV, Chiaramonti BC, Biruel E, de Castro Pochini A, Ejnisman B, Cohen M. Epidemiology of sports injuries in basketball: integrative systematic review. BMJ Open Sport Exerc. 2018;4(1). 10.1136/bmjsem-2018-000468 PMC6326319 30687514

[CR3] Ayala F, López-Valenciano A, Martín JA, Croix MD, Vera-Garcia FJ, del Pilar G-V, Ruiz-Pérez I, Myer GD (2019). A preventive model for hamstring injuries in professional soccer: Learning algorithms. Int J Sports Med.

[CR4] Bahr R, Clarsen B, Ekstrand J (2018). Why we should focus on the burden of injuries and illnesses, not just their incidence. Br J Sports Med.

[CR5] Bahr R, Clarsen B, Derman W, Dvorak J, Emery CA, Finch CF, et al. International Olympic Committee consensus statement: methods for recording and reporting of epidemiological data on injury and illness in sports 2020 (including the STROBE extension for sports injury and illness surveillance (STROBE-SIIS)). Orthop J Sports Med. 2020;8(2). 10.1177/2325967120902908 PMC7029549 32118084

[CR6] Bayne H, Schwellnus M, van Rensburg DJ, Botha J, Pillay L. Incidence of injury and illness in South African professional male soccer players: A prospective cohort study. J Sports Med Phys Fitness. 2018;58(6). 10.23736/S0022-4707.17.07452-7 28488835

[CR7] Bowen L, Gross AS, Gimpel M, Li FX (2017). Accumulated workloads and the acute: chronic workload ratio relate to injury risk in elite youth football players. Br J Sports Med.

[CR8] Brown JC, Cross M, England M, Finch CF, Fuller GW, Kemp SP (2019). Guidelines for community-based injury surveillance in rugby union. J Sci Med Sport.

[CR9] Chandran A, Nedimyer AK, Register-Mihalik JK, DiPietro L, Kerr ZY (2019). Comment on:“incidence, severity, aetiology and prevention of sports injuries: a review of concepts”. Sports Med.

[CR10] Chandran A, DiPietro L, Young H, Elmi A (2020). Modelling time loss from sports-related injuries using random effects models: an illustration using soccer-related injury observations. J Quant Anal Sports.

[CR11] Chandran A, Morris SN, Boltz AJ, Robison HJ, Collins CL (2021). Epidemiology of injuries in national collegiate athletic association men’s soccer: 2014–2015 through 2018–2019. J Athl Train.

[CR12] Chandran A, Morris SN, Boltz AJ, Robison HJ, Collins CL (2021). Epidemiology of injuries in national collegiate athletic association women’s soccer: 2014–2015 through 2018–2019. J Athl Train.

[CR13] Chandran A, Morris SN, Powell JR, Boltz AJ, Robison HJ, Collins CL (2021). Epidemiology of injuries in national collegiate athletic association men’s football: 2014–2015 through 2018–2019. J Athl Train.

[CR14] Chandran A, Boltz AJ, Morris SN, Robison HJ, Nedimyer AK, Collins CL (2022). Epidemiology of concussions in National Collegiate Athletic Association (NCAA) sports: 2014/15-2018/19. Am J Sports Med.

[CR15] Chandran A, Boltz AJ, Brett BL, Walton SR, Robison HJ, Collins CL, et al. Patterns and predictors of concussion symptom presentations in NCAA athletes. Res Sports Med. 2022;1–5. 10.1080/15438627.2022.2105218 35916338

[CR16] Dreyer NA, Mack CD, Anderson RB, Wojtys EM, Hershman EB, Sills A (2019). Lessons on data collection and curation from the NFL injury surveillance program. Sports Health.

[CR17] Edwards P, Roberts I, Clarke M, DiGuiseppi C, Pratap S, Wentz R (2002). Increasing response rates to postal questionnaires: systematic review. Br Med J.

[CR18] Ekstrand J (2013). Keeping your top players on the pitch: the key to football medicine at a professional level. Br J Sports Med.

[CR19] Ekstrand J, Bengtsson H, Waldén M, Davison M, Khan KM, Hägglund M (2023). Hamstring injury rates have increased during recent seasons and now constitute 24% of all injuries in men’s professional football: the UEFA Elite Club Injury Study from 2001/02 to 2021/22. Br J Sports Med.

[CR20] Emery CA, Meeuwisse WH, Hartmann SE (2005). Evaluation of risk factors for injury in adolescent soccer: implementation and validation of an injury surveillance system. Am J Sports Med.

[CR21] Finch C (2006). A new framework for research leading to sports injury prevention. J Sci Med Sport.

[CR22] Fuller CW, Ekstrand J, Junge A (2006). Consensus statement on injury definitions and data collection procedures in studies of football (soccer) injuries. Clin J Sport Med.

[CR23] Fuller CW, Molloy MG, Bagate C, Bahr R, Brooks JH, Donson H (2007). Consensus statement on injury definitions and data collection procedures for studies of injuries in rugby union. Br J Sports Med.

[CR24] Gardner AJ, Quarrie KL, Iverson GL (2019). The epidemiology of sport-related concussion: what the rehabilitation clinician needs to know. J Orthop Sports Phys Ther.

[CR25] Gould HP, Lostetter SJ, Samuelson ER, Guyton GP (2023). Lower extremity injury rates on artificial turf versus natural grass playing surfaces: a systematic review. Am J Sports Med.

[CR26] Graham MR, Myers T, Evans P, Davies B, Cooper SM, Bhattacharya K (2011). Direct hits to the head during amateur boxing is associated with a rise in serum biomarkers for brain injury. Int J Immunopathol Pharmacol.

[CR27] Griffin A, Kenny IC, Comyns TM, Lyons M (2020). The association between the acute: chronic workload ratio and injury and its application in team sports: a systematic review. Sports Med.

[CR28] Hägglund M, Waldén M, Ekstrand J (2006). Previous injury as a risk factor for injury in elite football: a prospective study over two consecutive seasons. Br J Sports Med.

[CR29] Halperin I, Vigotsky AD, Foster C, Pyne DB (2018). Strengthening the practice of exercise and sport-science research. Int J Sports Physiol Perform.

[CR30] Hassan J, Shehzad D, Habib U, Aftab MU, Ahmad M, Kuleev R, et al. The rise of cloud computing: data protection, privacy, and open research challenges—a systematic literature review (SLR). Comput Intell Neurosci. 2022;2022. 10.1155/2022/8303504 PMC9197654 35712069

[CR31] Horan D, Blake C, Hägglund M (2022). Injuries in elite-level women’s football—a two-year prospective study in the Irish women’s national league. Scand J Med Sci Sports.

[CR32] Jones A, Jones G, Greig N, Bower P, Brown J, Hind K (2019). Epidemiology of injury in English professional football players: a cohort study. Phys Ther Sport.

[CR33] King DA, Gabbett TJ, Gissane C, Hodgson L (2009). Epidemiological studies of injuries in rugby League: suggestions for definitions, data collection and reporting methods. J Sci Med Sport.

[CR34] Lee JW, Mok KM, Chan HC, Yung PS, Chan KM (2014). A prospective epidemiological study of injury incidence and injury patterns in a Hong Kong male professional football league during the competitive season. Asia-Pac J Sports Med Arthrosc.

[CR35] Lee EC, Fragala MS, Kavouras SA, Queen RM, Pryor JL, Casa DJ (2017). Biomarkers in sports and exercise: tracking health, performance, and recovery in athletes. J Strength Cond Res.

[CR36] Lempke LB, Chandran A, Boltz AJ, Robison HJ, Collins CL, Morris SN (2021). Epidemiology of injuries in national collegiate athletic association women’s basketball: 2014–2015 through 2018–2019. J Athl Train.

[CR37] Mack CD, Kent RW, Coughlin MJ, Shiue KY, Weiss LJ, Jastifer JR (2020). Incidence of lower extremity injury in the national football league: 2015 to 2018. Am J Sports Med.

[CR38] Mack CD, Solomon G, Covassin T, Theodore N, Cárdenas J, Sills A (2021). Epidemiology of concussion in the national football league, 2015–2019. Sports Health.

[CR39] Maniar N, Carmichael DS, Hickey JT, Timmins RG, San Jose AJ, Dickson J (2023). Incidence and prevalence of hamstring injuries in field-based team sports: a systematic review and meta-analysis of 5952 injuries from over 7 million exposure hours. Br J Sports Med.

[CR40] McBurnie AJ, Harper DJ, Jones PA, Dos’ Santos T. Deceleration training in team sports: Another potential ‘vaccine’for sports-related injury?. Sports Med. 2022;1–2. 10.1007/s40279-021-01583-x PMC8761154 34716561

[CR41] McGroarty NK, Brown SM, Mulcahey MK. Sport-related concussion in female athletes: a systematic review. Orthop J Sports Med. 2020;8(7). 10.1177/2325967120932306 PMC7366411 32728590

[CR42] McKay AK, Stellingwerff T, Smith ES, Martin DT, Mujika I, Goosey-Tolfrey VL (2021). Defining training and performance caliber: a participant classification framework. Int J Sports Physiol Perform.

[CR43] Mooney J, Self M, ReFaey K, Elsayed G, Chagoya G, Bernstock JD, Johnston JM (2020). Concussion in soccer: a comprehensive review of the literature. Concussion.

[CR44] Morgan BE, Oberlander MA (2001). An examination of injuries in major league soccer: the inaugural season. Am J Sports Med.

[CR45] Orchard J, Rae K, Brooks J, Hägglund M, Til L, Wales D (2010). Revision, uptake and coding issues related to the open access orchard sports injury classification system (OSICS) versions 8, 9 and 10.1. Open Access J Sports Med.

[CR46] Orchard JW, Ranson C, Olivier B, Dhillon M, Gray J, Langley B (2016). International consensus statement on injury surveillance in cricket: a 2016 update. Br J Sports Med.

[CR47] Pierpoint LA, Caswell SV, Walker N, Lincoln AE, Currie DW, Knowles SB (2019). The first decade of web-based sports injury surveillance: descriptive epidemiology of injuries in US high school girls’ lacrosse (2008–2009 through 2013–2014) and National Collegiate Athletic Association women’s lacrosse (2004–2005 through 2013–2014). J Athl Train.

[CR48] Rommers N, Rössler R, Verhagen E, Vandecasteele F, Verstockt S, Vaeyens R (2020). A machine learning approach to assess injury risk in elite youth football players. Med Sci Sports Exerc.

[CR49] Shahim P, Tegner Y, Wilson DH, Randall J, Skillbäck T, Pazooki D (2014). Blood biomarkers for brain injury in concussed professional ice hockey players. JAMA Neurol.

[CR50] Sprouse B, Alty J, Kemp S, Cowie C, Mehta R, Tang A, et al. The football association injury and illness surveillance study: the incidence, burden and severity of injuries and illness in men’s and women’s international football. Sports Med. 2020;1–20. 10.1007/s40279-020-01411-8 PMC7768595 33369724

[CR51] Stubbe JH, Van Beijsterveldt AM, Van Der Knaap S, Stege J, Verhagen EA, Van Mechelen, et al. Injuries in professional male soccer players in the Netherlands: a prospective cohort study. J Athl Train. 2015;50(2):211–6. 10.4085/1062-6050-49.3.64 PMC4495432 25531144

[CR52] Svoboda SJ, Harvey TM, Owens BD, Brechue WF, Tarwater PM, Cameron KL (2013). Changes in serum biomarkers of cartilage turnover after anterior cruciate ligament injury. Am J Sports Med.

[CR53] Tremblay MS (2018). The consequences of sedentary behaviors: keeping interpretations anchored in evidence. Exerc Sport Sci Rev.

[CR54] Van Eetvelde H, Mendonça LD, Ley C, Seil R, Tischer T (2021). Machine learning methods in sport injury prediction and prevention: a systematic review. J Exp Orthop.

[CR55] van Mechelen W, Hlobil H, Kemper HC (1992). Incidence, severity, aetiology and prevention of sports injuries. A review of concepts. Sports Med.

[CR56] Verhagen E, Bolling C (2015). Protecting the health of the@ hlete: how online technology may aid our common goal to prevent injury and illness in sport. Br J Sports Med.

[CR57] Waldén M, Mountjoy M, McCall A, Serner A, Massey A, Tol JL, et al. Football-specific extension of the IOC consensus statement: methods for recording and reporting of epidemiological data on injury and illness in sport 2020. Br J Sports Med. 2023. 10.1136/bjsports-2022-106405 PMC10646851 36609352

[CR58] Wasserman EB, Register-Mihalik JK, Sauers EL, Currie DW, Pierpoint LA, Knowles SB (2019). The first decade of web-based sports injury surveillance: descriptive epidemiology of injuries in US high school girls’ softball (2005–2006 through 2013–2014) and national collegiate athletic association women’s softball (2004–2005 through 2013–2014). J Athl Train.

[CR59] Yard EE, Collins CL, Comstock RD (2009). A comparison of high school sports injury surveillance data reporting by certified athletic trainers and coaches. J Athl Train.

