# Supplementary figures and images for: Injury and illness surveillance monitoring in team sports: a framework for all
Source: Inj Epidemiol. 2024 Jun 10;11:23. doi: 10.1186/s40621-024-00504-6 (PMC11163858; doi:10.1186/s40621-024-00504-6)

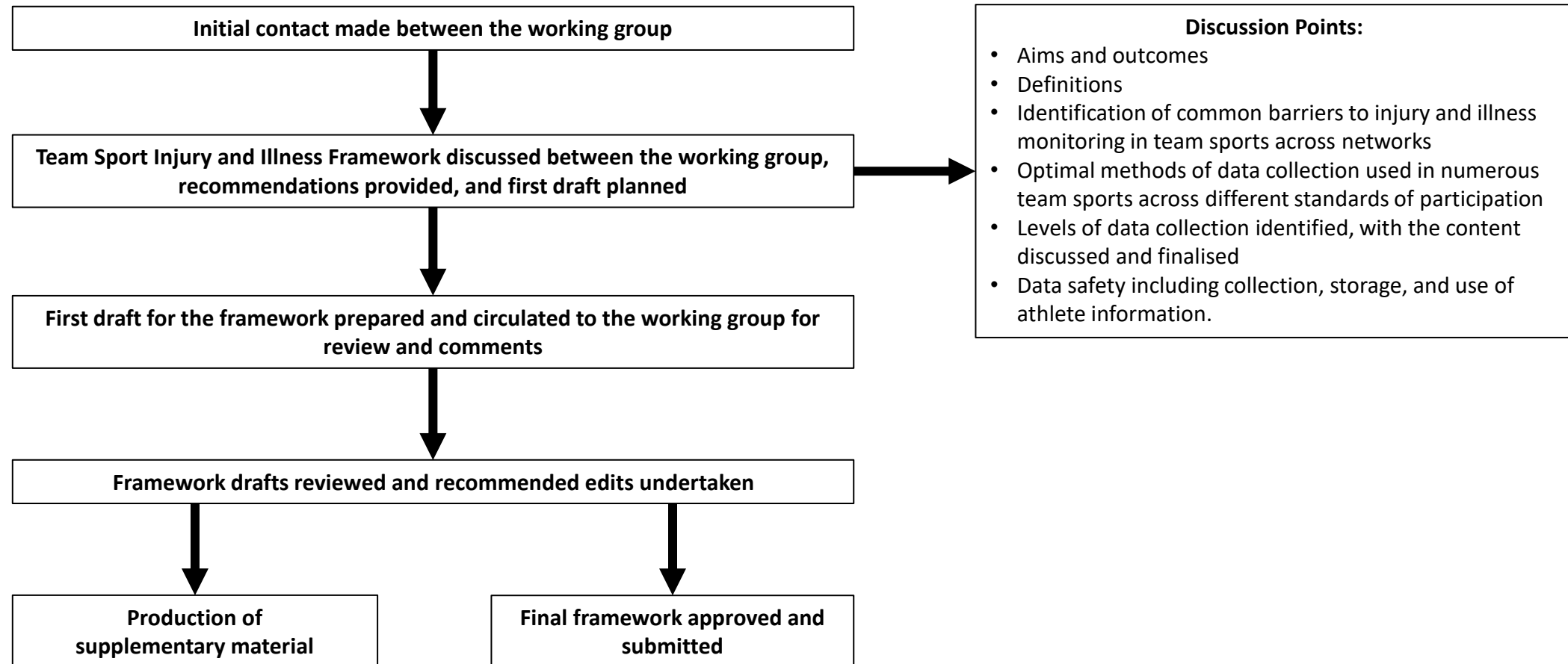

Supplement: Supplementary file 1 — Additional file 1. Workflow diagram [file 40621_2024_504_MOESM1_ESM.pdf]
